# Truths and Lies from the Polysomnography ECG Recording: An Electrophysiologist Perspective

**DOI:** 10.1155/2009/675078

**Published:** 2009-05-04

**Authors:** Adrian Baranchuk, Christina Quinlan, Kevin Michael, Christopher S. Simpson, Damian P. Redfearn, Michael Fitzpatrick

**Affiliations:** Cardiac Electrophysiology and Pacing, Kingston General Hospital, Queen's University, ON, Canada K7L 2V7

## Abstract

Polysomnography remains the gold standard for diagnosis of Sleep Apnea (SA) and evaluation of the apnea/hypopnea index (AHI) which is used as the primary index of SA severity. The electrocardiogram (typically a single lead) obtained during the polysomnographic study is usually used to report the association between SA and cardiac rhythm disturbances. These findings help in guiding medical decisions but they could also represent a source for confusion. Electrophysiologists are frequently consulted to determine whether interventions need to be taken. We present 2 cases where the ECG during a polysomnography study required the intervention of an electrophysiologist to help with management.

## 1. Introduction

Polysomnography remains the gold standard for diagnosis of Sleep Apnea (SA) and evaluation of the AHI, the primary index of Sleep Apnea severity [1]. It typically consists of simultaneous recording of electroencephalography, electrooculography, electromyography, electrocardiogram (ECG), right and left anterior tibialis EMG, oxygen saturation, as well as oronasal airflow; and thoracic and abdominal movement and body position via infrared video monitoring. The ECG (frequently a single lead) obtained during the study is usually used to report the cardiac rate and rhythm and any association between SA and cardiac rhythm disturbances. These findings help in guiding medical decisions but they could also represent a source of confusion. Electrophysiologists are frequently consulted to determine whether interventions need to be taken.

**Case 1**

A 67-year-old man with history of lightheadedness and hypertension was referred to the Sleep Disorder Clinic. The patient was on no antiarrhythmic drugs and was taking hydrochlorothiazide 50 mg/d, fosinopril 40 mg/d and amlodipine 10 mg/d for blood pressure control.

**Figure 1:**
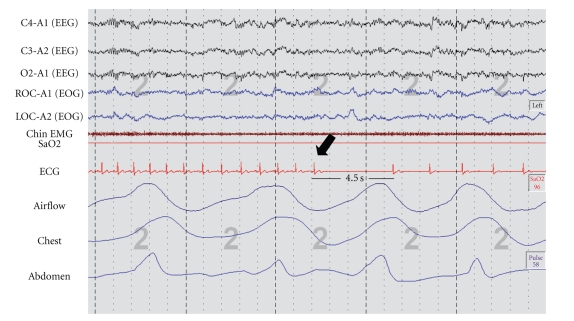
25 seconds snap shot from stage 2 non-REM study in patient number 1. Black arrow shows the onset of sinus arrest lasting 4.5 seconds. After the pause, junctional rhythm is observed. Upper channels (C4-A1) (C3-A2) (O2-A1): electroencephalogram; fourth and fifth channel (ROC-A1) (LOC-A2): oculogram; sixth channel: chin electromyogram; seventh channel: oxygen saturation; eigth channel: electrocardiogram; ninth channel: airflow; tenth channel: chest movement; eleventh channel: abdomen movement.

The sleep study showed no evidence of SA but several pauses due to sinus arrest were observed (Figure 1, black arrow). The longest pause lasted for 7 seconds. The pauses were not preceded nor followed by apneic episodes. The patient was referred to the Arrhythmia Service, and Holter monitoring was performed. Several diurnal sinus node pauses longer than 5 seconds were detected. A pacemaker was implanted which resulted in total resolution of the symptoms at 18 months followup.

**Case 2**

A 58-year-old train driver with excessive daily sleepiness, fatigue, and frequent snoring was referred to the Sleep Disorder Clinic. The patient was on no antiarrhythmic drugs and receiving ramipril 20 mg/d for blood pressure control.

**Figure 2:**
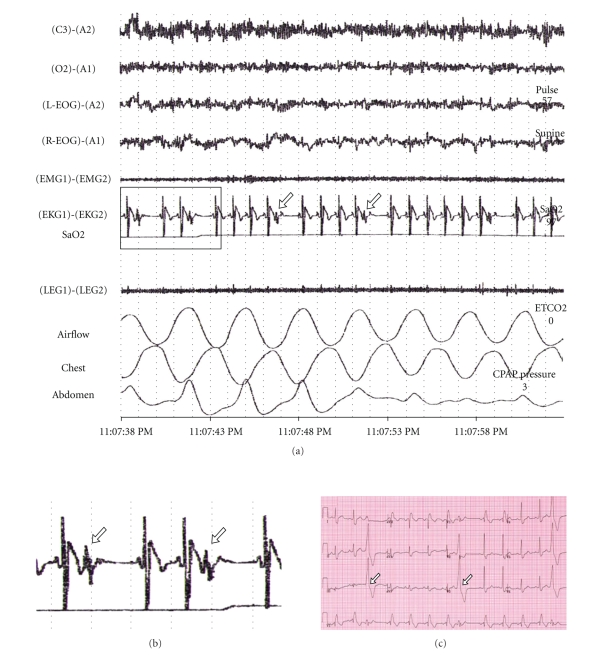
(a) 25 seconds snap shot from sleep study in patient number 2 during wakefulness. White arrows show the beats that were initially reported as non-conducted P-waves. Upper channels (C3-A1) (01-A1): electroencephalogram; third and fourth channel (L-EOG-A1) (R-EOG-A1): oculogram; fifth channel (EMG1) (EMG2): electromyogram; sixth channel (EKG1) (EKG2): electrocardiogram; seventh channel (SaO2): oxygen saturation; eighth channel (LEG1) (LEG2): leg movement; ninth channel: airflow; tenth channel: chest movement; eleventh channel: abdomen movement. (Panel (b)) Amplification of the area under the highlighted rectangle in panel (a). White arrows show the PVCs followed by a post-extrasystolic pause. (Panel (c)) 12-lead ECG shows right bundle branch block. White arrows show PVCs arising probably from the left outflow tract.

The study showed moderate obstructive SA with AHI of 17. The ECG of the polysomnography was reported as second degree AV block Mobitz II (Figure 2(a)). The patient was referred to the Arrhythmia Service for consideration of pacemaker insertion.

A closer look to the ECG recording suggests the presence of ectopic beats followed by a postextrasystolic pause rather than what were considered to be nonconducted P-waves (Figure 2(b), white arrows). A 12-lead ECG was obtained showing right bundle branch block and frequent premature ventricular contractions (PVC) with a possible left ventricular outflow tract origin (Figure 2(c), white arrows). The PR interval and the QRS width in sinus rhythm measured 178 ms and 176 ms, respectively.

An event monitor showed isolated PVCs with fixed coupling interval.

The patient was discharged from the Arrhythmia Service without specific recommendations and was encouraged to initiate C-PAP for treating SA.

## 2. Discussion

These 2 cases represent fairly typical and common consultation requests for the cardiac electrophysiologist. Sometimes the single lead ECG recording during polysomnography may help uncover a conduction disorder that requires specific cardiac intervention, as in case 1. However, on other occasions, this single lead recording may be difficult to interpret as in case 2.

Several case reports have linked SA to bradyarrhythmias in the past [2]. However, the largest epidemiological study to date has failed to demonstrate a significant association between these two conditions [3]. The main hypothesis supporting the association of SA and bradyarrhythmias is based on autonomic imbalance produced by the recurrent apnea and repetitive episodes of hypoxemia [4, 5].

Several consults arrive to the arrhythmia service to evaluate patients with SA and suspected conduction disorders for possible cardiac pacing. It is clear that some patients may require a pacemaker insertion to treat the bradyarrhythmia that may or may not be associated with SA, and which does not infer that the SA will improve with cardiac pacing.

In the first case presented in this paper, the patient had no clear indication of sleep apnea; however, the ECG recording during polysomnography was the key finding that led to further appropriate investigation and treatment. The Holter monitoring not only confirmed the presence of diurnal pauses but also permitted symptom-rhythm correlation. After implant, complete resolution of symptoms occurred, suggesting that bradycardia was the cause of symptoms.

The second case was more challenging because a conduction disorder was diagnosed at the sleep disorder clinic orienting this patient towards device implantation. The initial analysis may be confusing with small waves suggesting the presence of non-conducted P-waves. A closer look and 12-lead ECG permitted one to determine the presence of a premature QRS followed by a compensatory post-extrasystolic pause. This matches with the PVCs observed in the 12-lead ECG. Given the absence of symptoms suggesting paroxysmal AV block and the finding of isolated PVCs in a structurally normal heart, the decision was to recommend SA treatment without further electrophysiologic investigations.

## 3. Conclusions

It is of the utmost importance for electrophysiologists to become familiar with ECG tracings during polysomnography study. In cases such as those described herein, proper polysomnography ECG tracing interpretation may help in the decision-making process regarding pacemaker implantation.
